# Vascular endothelial growth factor directly stimulates tumour cell proliferation in non-small cell lung cancer

**DOI:** 10.3892/ijo.2015.3082

**Published:** 2015-07-14

**Authors:** AOIFE M. DEVERY, REKHA WADEKAR, SIVAN M. BOKOBZA, ANIKA M. WEBER, YANYAN JIANG, ANDERSON J. RYAN

**Affiliations:** CRUK and MRC Oxford Institute for Radiation Oncology, Department of Oncology, University of Oxford, Headington, Oxford OX3 7DQ, UK

**Keywords:** vascular endothelial growth factor, VEGFR2, cediranib, AZD6244, MK2206, lung cancer, non-small cell lung cancer

## Abstract

Vascular endothelial growth factor (VEGF) is a key stimulator of physiological and pathological angiogenesis. VEGF signals primarily through VEGF receptor 2 (VEGFR2), a receptor tyrosine kinase whose expression is found predominantly on endothelial cells. The purpose of this study was to determine the role of VEGFR2 expression in NSCLC cells. NSCLC cells and tissue sections were stained for VEGFR2 expression by immunohistochemistry (IHC). Immunoblotting and ELISA were used to determine the activation and inhibition of VEGFR2 and its downstream signalling pathways. Five-day proliferation assays were carried out in the presence or absence of VEGF. IHC analysis of NSCLC demonstrated tumour cell VEGFR2 expression in 20% of samples. Immunoblot analysis showed expression of VEGFR2 protein in 3/8 NSCLC cell lines that correlated with VEGFR2 mRNA expression levels. VEGF-dependent VEGFR2 activation was apparent in NSCLC cells, and was associated with increased tumor cell proliferation. Cediranib treatment or siRNA against VEGFR2 inhibited VEGF-dependent increases in cell proliferation. Inhibition of VEGFR2 tyrosine kinase activity using cediranib was more effective than inhibition of AKT (MK2206) or MEK (AZD6244) for overcoming VEGFR2-driven cell proliferation. VEGF treatment did not affect cell survival following treatment with radiation, cisplatin, docetaxel or gemcitabine. Our data suggest that a subset of NSCLC tumour cells express functional VEGFR2 which can act to promote VEGF-dependent tumour cell growth. In this tumour subset, therapies targeting VEGFR2 signalling, such as cediranib, have the potential to inhibit both tumour cell proliferation and angiogenesis.

## Introduction

Neovascularization of solid tumours plays an important role in tumour cell growth and metastasis (1). Although numerous growth factors and cytokines stimulate angiogenesis, vascular endothelial growth factor (VEGF) plays the predominant role in stimulating neovascularization (1). VEGF is overexpressed by a majority of solid tumours, and circulating levels of VEGF are elevated in many cancer patients, including lung cancer (2). Activation of VEGF receptor (primarily VEGFR2) downstream signalling pathways by VEGF increases vascular permeability and promotes endothelial cell proliferation, survival and migration in both physiological and pathological angiogenesis (2).

Several approaches to inhibiting tumour angiogenesis by targeting VEGF signalling have been developed (3–6) and are currently approved for use in the clinic against a number of tumour types including colorectal (3), renal (5), glioblastoma (7), hepatocellular (8) and lung (9). However, identification of the patient subsets which responds to VEGF signalling inhibition remains elusive (10).

VEGFR2 protein has been reported to be expressed in cells of solid tumours including breast (10), gastrointestinal (11), prostate (7), melanoma (12,13) and non-small cell lung carcinoma (NSCLC) (14–19). In principal, the use of VEGF-signalling inhibitors in the treatment of these cancers might inhibit tumour angiogenesis and additionally reduce tumour cell proliferation, invasion and survival.

The role of VEGFR2 protein expression in NSCLC has not yet been elucidated. The aim of this work is to investigate the role of VEGFR2 in NSCLC cell lines and the potential impact of signalling inhibition.

## Materials and methods

### Materials

Recombinant human VEGF165 (R&D Systems, Abingdon, UK) was prepared in sterile dH_2_O. Cisplatin (Sigma, Dorset, UK) was prepared at 3.3 mM in PBS. Docetaxel, gemcitabine, pemetrexed (LC Laboratories, Woburn, UK) and the AKT inhibitor, MK2206, MEK inhibitor, AZD6244, and VEGFR inhibitor, Cediranib/AZD2171 (6) (Selleck, Suffolk, UK), were prepared as 10 mM stocks in DMSO and stored at −20°C. Formalin-fixed tumour samples were obtained from ProteoGenex (Culver City, CA, USA). For radiation, cells were exposed to 10 Gy (^137^Cs, 1.958 Gy/min) in a Gamma services GSR-D1 irradiator. Hoechst 33258 (Sigma) was prepared in dH_2_O at 10 mg/ml and stored at 4°C.

### Cell lines

SKBR3 (Leibniz Institute DSMZ, Braunschweig, Germany), H3122 [National Cancer Institute (NCI), USA] and other cell lines (all from ATCC; Manassas, VA, USA), and were cultured in Advanced DMEM-F12 (Life Technologies, Paisley, UK) media with 5% foetal bovine serum (Sigma), 2 mM GlutaMAX (Life Technologies) and 50 units of penicillin/50 μg/ml streptomycin (Life Technologies) at 37°C with 7.5% CO_2_.

### Immunohistochemistry

Sections (5 μm) of formalin-fixed NSCLC cell line pellets (n=25) or normal lung (n=4) or NSCLC tumour (n=52) were incubated overnight with a rabbit polyclonal antibody (CST #2479) against human VEGFR2. Formalin-fixed paraffin-embedded tumour samples were obtained from ProteoGenex with written patient consent and institutional review board/independent ethics committee (IRB/IEC) approval. Sections (5 μm) were washed and incubated with horseradish peroxidase (HRP)-linked goat anti-rabbit IgG and then stained with diaminobenzidine (DAB). For cell lines, staining categories (0, +, ++, +++) were defined using cell line pellets with cells of known high (TT, +++), medium (H441, ++), low (H1792, +) and negative (Calu3, 0) VEGFR2 expression. For evaluable tumour samples (n=51), tumour cell VEGFR2 expression was scored as positive (1; moderate or strong staining) or negative (0; weak or no staining) as previously reported (15).

### Quantitative PCR

Total RNA was isolated from NSCLC cell lines using RNeasy (Qiagen, Venlo, The Netherlands) and reverse transcribed into cDNA using iScript (Bio-Rad, Hertfordshire, UK). VEGFR2 cDNA was amplified by RT-PCR (Applied Biosystems, Paisley, UK) and expression quantified by the ΔΔCt method using peptidylprolyl isomerase A (PPIA) as the control. Primer sequences were: VEGFR2 forward: TTT CGC CCG GCT CGA GG TGC, VEGFR2 reverse: CTA GGC AAA CCC ACA GAG GCG GC; PPIA forward: CGC CAC CGC CGA GGA AAA CCG, PPIA reverse: CTG CAA ACA GCT CAA AGG AGA CGC GG.

### Immunoblotting

Immunoblotting was carried out as previously described (20). For VEGF stimulation, 6-well plates with cells at 70% confluency were incubated in low serum conditions (0.2% FBS) ± cediranib (100 nM) overnight and then treated with VEGF (0–100 ng/ml) for 0–60 min. Protein was extracted using 50 mM Tris-HCl pH 7.6, 137 mM NaCl, 10% glycerol, 0.1% Igepal, 0.1% SDS, 50 mM NaF, 1 mM Na_3_VO_4_ and cocktail protease inhibitor (1 tab per 25 ml of lysis buffer) on ice for 10 min. Immunoblots were incubated overnight at 4°C with antibodies against total and phosphorylated VEGFR2 (CST, Hertfordshire, UK. 1:800 dilution), p42/44 MAPK (CST, 1:1,000 dilution), AKT (CST, 1:1,000 dilution), PARP (CST, 1:1,000 dilution) and PPIB (Abcam, Cambridge, UK; 1:2,000). After washing, blots were incubated for 40 min with anti-mouse or anti-rabbit LI-COR secondary antibodies (LI-COR, Cambridge, UK; 1:50,000 dilution) or with horse-radish peroxidase (HRP) conjugated goat anti-mouse (Thermo Scientific, Boston, MA, USA; 1:8,000 dilution) or anti-rabbit (Thermo Scientific; 1:6,000 dilution) for 1 h. Detection was carried out using the LI-COR, Odyssey or ECL substrate (Thermo Scientific).

### Phosphorylated VEGFR2 ELISA

Phosphorylated VEGFR2 levels in cell lysates were determined with the PathScan phospho-VEGFR-2 (Tyr1175) Sandwich ELISA Kit Cell (CST; UK) according to manufacturer's instructions.

### Cell proliferation assay

Subconfluent cells were trypsinised, washed once with PBS and seeded in 0.2% FBS DMEM/F12 media at a density of 1,000 to 4,000 cells per well in 96-well plates. The plates were incubated overnight at 37°C with 7.5% CO_2_ to allow cells to attach, and then treated with VEGF (0–100 ng/ml) ± drug and incubated for a further 5 days. Cells were then stained with crystal violet, allowed to dry and the dye eluted using glacial acetic acid. The absorbance was read at 590 nm using the POLARstar Omega plate reader.

### VEGFR2 siRNA transfection

Small interfering RNAs (siRNA) targeting siRNA control (ON TARGETplus control siRNA, 100 nM) and VEGFR2 (ONTARGET plus smartpool, 100 nM) (Dharmacon, Inc., UK) smartpools were transfected into H441 cells using DharmaFECT 2 reagent (4 μl/ml, Dharmacon, Inc.) according to manufacturer's instructions. For the proliferation assay end point, cells were incubated in the transfection media for 24 h. The transfection media was removed and replaced with 100 μl of fresh 0.2% FBS DMEM/F12. DMEM/F12 treated with ± FBS or VEGF was added and plates were incubated for a further 4 days. At this point the cells were stained with crystal violet, allowed to dry and eluted with glacial acetic acid. The absorbance was read at 590 nm using the POLARstar Omega plate reader.

### Cell death assay

Cells were trypsinised, suspended in phenol red free 10% DMEM/F12 media and seeded at a density of 10,000 to 40,000 cells/ml in 96-well plates. Plates were incubated at 37°C with 7.5% CO_2_ overnight and then treated with drug ± 100 ng/ml VEGF and incubated for a further 24 h. Hoechst 33258 (15 μM) was added and incubation continued in the dark at 37°C with 7.5% CO_2_ for 30 min. Images of each well were captured using the In Cell Analyzer 1000 and analysed using Analyzer 1000 software. Live cells were distinguished from dead cells by the intensity of Hoechst binding.

### Migration assay

Boyden chambers were placed in a 24-well plate and rinsed once with serum free media. Subconfluent cells were trypsinised, wash once with PBS and seeded in 0.2% FBS DMEM/F12 media at a density of 200,000 cells per 50 μl per chamber. Into each chamber 50 μl of 0.2% FBS media treated with of VEGF ± cediranib was added and 250 μl of 0.2% FBS media was added below the chamber. The plate was incubated at 37°C for 48 h, at which point the migrated cells were stained with crystal violet. Migrated cells were counted under an inverted microscope.

### Statistics

Statistical comparisons was carried our using the Student's t-test. Significance was set at P≤0.05.

## Results

### VEGFR2 expression in NSCLC tumour cells and cell lines

**Figure 1:**
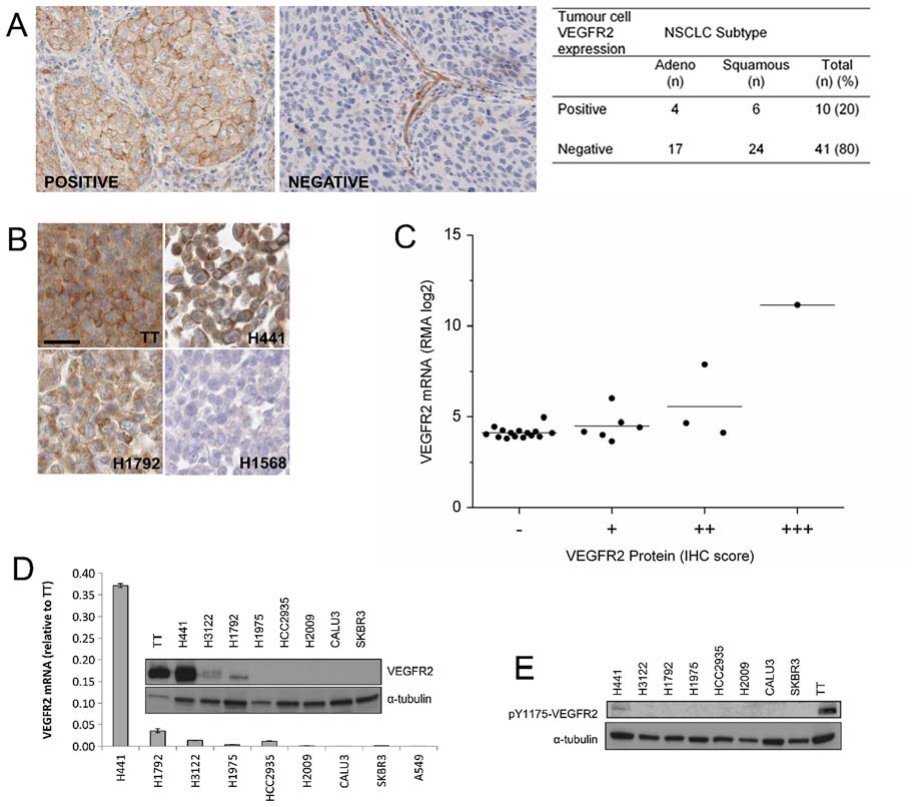
VEGFR2 protein is expressed in NSCLC tumours and tumour cell lines. (A) VEGFR2 protein expression in NSCLC by IHC. Examples of positive and negative tumour cell expression are shown. (B) Representative images of 3^+^ (TT, medullary thyroid cancer, positive control), 2^+^ (H441), 1^+^ (H1792) and negative (H1568) VEGFR2 staining in cell line pellets (bar 50 μm). (C) Relationship between VEGFR2 mRNA and protein levels in NSCLC cell lines (n=25) and a positive control cell line (TT). (D) Expression of total VEGFR2 protein by immunoblotting, and VEGFR2 mRNA levels by qPCR in a panel of NSCLC cell lines, including a positive control cell line (TT). (E) VEGF-stimulated activated (phosphorylated) VEGFR2 levels in the panel of NSCLC cell lines.

VEGFR2 expression in lung cancer cells remains unresolved (15,21–23). Using a validated antibody (22,23), we found VEGFR2 expression was present on the blood vessels of all tumour samples and in the tumour cells of 20% all samples (Fig. 1A). Expression was present in both adenocarcinoma and squamous cell carcinoma samples, but not in normal lung epithelial cells. In cell lines, VEGFR2 expression was seen in 9 out of 25 NSCLC cell lines by IHC and this was confirmed in 3 NSCLC lines by immunoblotting and RT-PCR (Fig. 1D). By immunoblotting, phosphorylation of VEGFR2 was also detectable in the H441 cells (Fig. 1E).

### VEGF stimulation of VEGFR2 phosphorylation in NSCLC cells

**Figure 2:**
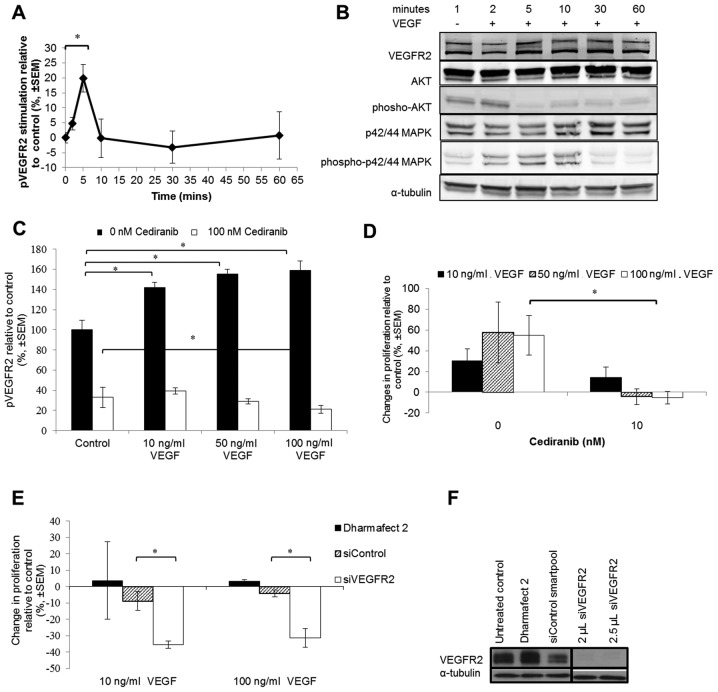
VEGFR2 activation by VEGF results in downstream phosphorylation of p42/44 MAPK and increased H441 proliferation. (A) pVEGFR2 levels measured by ELISA in serum-starved H441 cells following VEGF (10 ng/ml) stimulation. (B) Immunoblot of total and phosphorylated AKT and p42/44 MAPK following VEGF (10 ng/ml) stimulation of serum-starved H441 cells. (C) ELISA showing pVEGFR2 induction by VEGF and inhibition by cediranib in H441 cells. (D) Five-day proliferation assay for H441 in 0.2% FBS plus VEGF at 5 days in the presence of absence of cediranib or (E) VEGFR2 mRNA knockdown with siRNA. (F) VEGFR2 protein expression in H441 cells following treatment with transfection reagent alone (Dharmafect), control siRNA or VEGFR2 siRNA. Data are from three independent experiments, each in triplicate. ^*^P<0.05.

Immunoblotting against total VEGFR2 and quantitative ELISA against phospho-VEGFR2 showed that, after VEGF treatment, phosphorylation of VEGFR2 Y1175 was increased after 2 min, peaked at 5 min and had returned to baseline levels by 10 min (Fig. 2A). Immunoblotting against downstream proteins showed that AKT phosphorylation was reduced after 2 min but that p42/44 MAPK was significantly increased from 2 to 10 min (Fig. 2B). This activation corresponded with the phosphorylation of VEGFR2. The addition of cediranib prior to VEGF stimulation prevented or reduced the phosphorylation levels of VEGFR2 in the H441 cells (Fig. 2C).

### Activation of VEGFR2 signalling is associated with increased NSCLC cell proliferation

VEGF-induced phenotypic effects were first evaluated by proliferation assays (Fig. 2D and E). VEGF treatment over a 5-day period resulted in a 20–60% increase in H441 cell proliferation relative to the untreated control (Fig. 2D). Noteworthy, the level of VEGF-stimulation of H441 tumour cell proliferation was similar to the 35–100% increase in VEGF-stimulated endothelial cell proliferation previously reported (24,25). The addition of cediranib (a VEGFR tyrosine kinase inhibitor) prevented this increase in VEGF-stimulated cell proliferation (Fig. 2D). There was no effect of cediranib in H2009 or H1975 NSCLC cell lines which did not express VEGFR2 (data not shown). To validate these results we used VEGFR2 siRNA (Fig. 2F) which also reduced VEGF-stimulated H441 cell proliferation (Fig. 2E) suggesting that VEGF-induced cell proliferation is via VEGFR2 signalling.

### Radiation or drug-induced apoptosis are not affected by stimulation of VEGFR2

**Figure 3:**
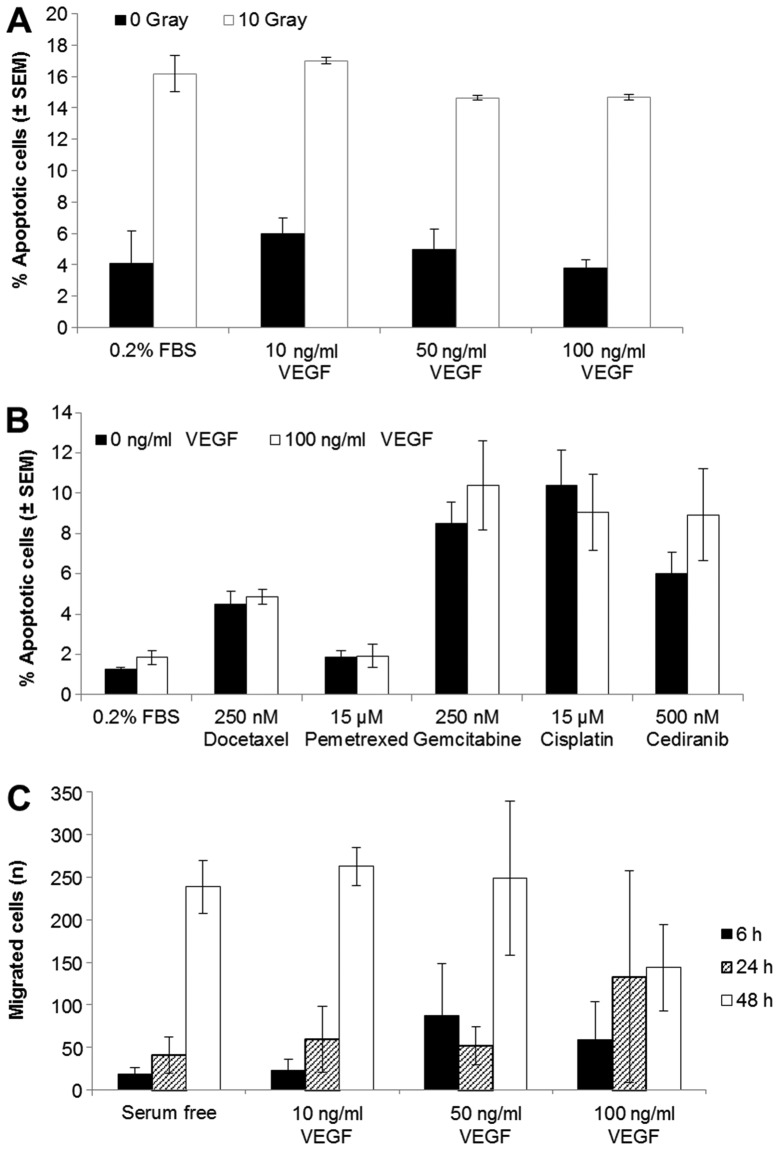
VEGF does not protect H441 cells from irradiation- or chemotherapy-induced apoptosis, or affect H441 migration. Induction of apoptosis in H441 cells following exposure to (A) irradiation or (B) cediranib or chemotherapy drugs relevant to NSCLC for 24 h in the presence or absence of VEGF. (C) VEGF-induced migration of serum-starved H441. Data are from three independent experiments, each in triplicate.

When we exposed VEGFR2 stimulated cells to irradiation (10 Gy, Fig. 3A) or to docetaxel, pemetrexed, gemcitabine, cisplatin or cediranib (Fig. 3B) there was no decrease in cell apoptosis compared with unstimulated cells. H441 cells treated with radiation had a 12% increase in apoptotic cells compared to the untreated cells. The addition of 10, 50 or 100 ng/ml VEGF 4 h prior to radiation did not reduce this induction of cell death (Fig. 3A). The chemotherapeutic drugs also increased the percentage of apoptotic cells relative to the untreated control after 24 h; however, VEGF-stimulated signalling did not prevent this increase (Fig. 3B).

### Effect of VEGFR2 activation on tumour cell migration

To determine if VEGFR2 signalling plays a role in NSCLC cell migration, we carried out migration assays of H441 cells in the presence or absence of VEGF (10, 50 or 100 ng/ml) (Fig. 3C). There was an increase in migration over a 48 h period; however, the stimulation of VEGFR2 signalling did not alter the migration of these cells. Wound healing (scratch) assays were also carried out under the same conditions but there was no increase in wound repair in the presence of VEGF (data not shown).

### Inhibition of VEGFR2 kinase activity or downstream signalling

**Figure 4:**
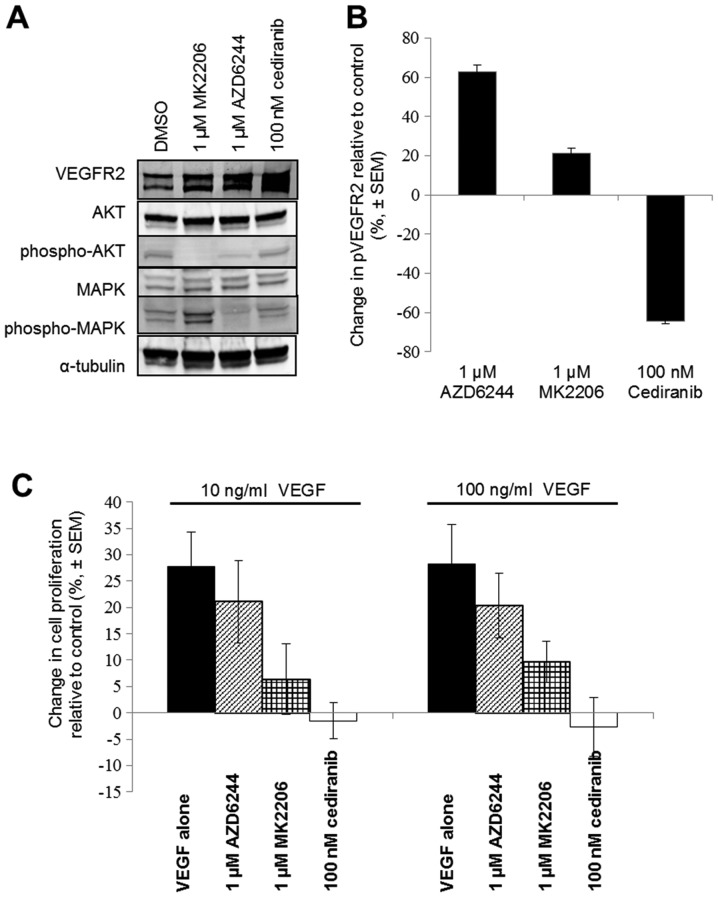
Targeting downstream signalling is not as effective as VEGFR2 kinase inhibition for checking VEGF-dependent H441 cell proliferation. (A) Immunoblot and (B) pVEGFR2 ELISA to evaluate the effects of 24 h treatment with a selective MEK inhibitor (AZD6244), AKT inhibitor (MK2206), or VEGFR inhibitor (cediranib). (C) The impact of AKT, MEK or VEGFR inhibition on VEGF stimulated proliferation in serum-starved H441 cells. Proliferation data are from three independent experiments, each in quadruplicate.

Immunoblotting lysates of H441 cells treated with cediranib, MK2206 (AKT inhibitor) or AZD6244 (MEK inhibitor) for 24 h showed that MK2206 significantly reduced AKT phosphorylation and AZD6244 reduced p42/44 MAPK phosphorylation and, to a lesser extent, AKT phosphorylation (Fig. 4A). Cediranib treatment did not produce a sustained reduction in AKT or p42/44 MAPK phosphorylation after 24 h (Fig. 4A), however only cediranib maintained a reduction in phosphorylated levels of VEGFR2 (Fig. 4B). In the proliferation assays, cediranib was more effective than the AKT inhibitor or MEK inhibitor (Fig. 4C) at reducing or preventing VEGF stimulated VEGFR2 proliferation.

## Discussion

It is well established that VEGFR2 activation plays a pivotal role in increased vascular permeability and in EC proliferation, migration and invasion (26). VEGFR2 protein expression has also been reported in tumour cells of haematological and solid tumours including breast, colon, prostate and melanoma, where roles in tumour cell proliferation, survival and migration were reported (7,10–12,27,28). While VEGFR2 expression has been reported in lung cancer by some groups (15,21,23,29,30); the clinical relevance of this expression remains uncertain (14,19,31). Others have reported that VEGFR2 is not expressed in NSCLC (22).

We confirmed the expression of VEGFR2 protein in NSCLC tumour samples by IHC using a validated antibody (22,23) and confirmed VEGFR2 mRNA and protein expression in a subset of NSCLC cell lines. The kinetics of VEGF-dependent VEGFR2 and MAPK phosphorylation in NSCLC cells was consistent with other studies in ECs (32,33) suggesting VEGF activation of VEGFR2 signalling may be similar in both cell types. However, in contrast with previous work (30,34), our data suggest that VEGFR2 activation in NSCLC cells is associated with a rapid decrease in pAKT. Contrasting effects of VEGFR2 signalling on tumour cell proliferation have been reported, with some studies suggesting increased tumour cell proliferation (7,29) whereas others suggest inhibition (35,36).

In our study, VEGFR2 TK (cediranib) fully inhibited VEGF-dependent VEGFR2 proliferation in NSCLC cells, whereas MAPK inhibitor (AZD6244) was not effective. AKT inhibitor (MK2206) partially reduced VEGF-dependent proliferation although this was not associated with a reduction in pVEGFR2. Of note, both pAKT and pMAPK (but not pVEGFR2) levels returned to control levels within 24 h of treatment with cediranib, but not with MK2206 or AZD6244, respectively. This suggests that the activity of MAPK and AKT signalling pathways are not directly driving VEGFR2-dependent proliferation in NSCLC. VEGFR2-dependent changes in pAKT have not been reported for other solid tumour types so the importance of this decrease in pAKT in NSCLC is not known. We also found that cediranib significantly reduced VEGFR2 phosphorylation in untreated NSCLC cells indicating a potential functional autocrine VEGF/VEGFR2 signalling loop as previously suggested for melanoma (27).

We were not able to demonstrate a significant role for VEGF-dependent VEGFR2 signalling in NSCLC cell migration or survival following treatment with radiation or cytotoxic agents. This suggests a more restricted role for VEGFR2 signalling in NSCLC than the roles that have been reported in ECs and other tumour types (29). AKT is recognized as a general mediator of survival signals following exposure to cytotoxic agents (37). However, in our studies on NSCLC cells, VEGF-stimulation did not increase AKT signalling and VEGFR2 inhibition did not reduce pAKT levels, which could underlie the lack of effect on survival.

VEGFR2-dependent increases in pMAPK have been reported for carcinoid and SCLC, and were associated with increases in tumour cell migration (29,35). However, in our studies, VEGFR2 expressing NSCLC cells were poorly migratory in both Boyden chambers and scratch wound assays and therefore we were unable to demonstrate a significant effect of exogenous VEGF in these assays. Although our study shows an acute increase of pMAPK levels following VEGF stimulation, this returned to baseline levels within 30 min suggesting MAPK pathway activity may not be sustained sufficiently to produce a migratory phenotype.

Inhibition of VEGF signalling markedly inhibits tumour growth of human tumour xenografts from a broad range of solid tumour types, including lung cancer, suggesting that inhibition of angiogenesis is the dominant mode of action in mouse models (30,38). Conversely, in unselected patients in human disease, VEGFR inhibitors as single agents show only modest activity in NSCLC (39–41) but greater activity in other tumour settings (5,7,8), perhaps suggesting that non-anti-angiogenic modes of action may contribute more significantly to activity in lung cancer in certain patients in the clinic.

Currently, there are no biomarkers that can identify the patients that respond to VEGF-signalling inhibitors. The majority of solid tumours express VEGF and levels are known to increase with tumour stage (17) and to correlate with shorter time to tumour progression and poor prognosis (2). In addition, high tumour cell VEGFR2 expression in NSCLC has been shown to be associated with poor prognosis (19,31). To our knowledge, this is the first study of a VEGF/VEGFR2 dependent cell proliferation pathway in NSCLC, with potential to drive tumour growth through an autocrine signalling pathway. An autocrine VEGF/VEGFR2 signalling loop has been reported in NSCLC cells acting to stimulate VEGF production and angiogenesis (42).

In conclusion, collectively, our results suggest that patients with NSCLC whose tumour cells express high levels of VEGFR2 may have tumour growth stimulated through both angiogenesis and increased proliferation. As a consequence, VEGFR2-expressing NSCLC tumours may be particularly sensitive to VEGFR2 TKIs through two distinct effects of VEGF. Our data also suggest that VEGFR2 expression in tumour cells is biologically relevant, and is a potential biomarker to identify NSCLC patients who may gain the greatest benefit from anti-VEGFR2 therapy.

## Abbreviations

VEGF: vascular endothelial growth factor
VEGFR2: vascular endothelial growth factor receptor 2
SCLC: small cell lung carcinoma
NSCLC: non-small cell lung carcinoma
IHC: immunohistochemistry
TKI: tyrosine kinase inhibitor
EC: endothelial cells
